# Will the Higher-Income Country Blueprint for COVID-19 Work in Low- and Lower Middle-Income Countries?

**DOI:** 10.9745/GHSP-D-20-00217

**Published:** 2020-06-30

**Authors:** Stephen Hodgins, Abdulmumin Saad

**Affiliations:** a Editor-in-Chief, Global Health: Science and Practice Journal, and Associate Professor, School of Public Health, University of Alberta, Edmonton, Alberta, Canada.; b Global Health Support Initiative-III, United States Agency for International Development, Washington, DC, USA.

## Abstract

Strategies to radically suppress incidence of COVID-19, as used in higher-income countries, may be unrealistic and counterproductive in most low- and lower middle-income countries. Instead, strategies should be tailored to the setting, balancing expected benefits, potential harms, and feasibility.

## Key Message

Strategies currently pursued in high-income and upper middle-income countries—aimed at radically suppressing incidence of COVID-19—may be unrealistic and counterproductive in most low- and lower middle-income countries. Instead, strategies need to be tailored to the setting, balancing expected benefits, potential harms, and feasibility.

## BACKGROUND

The Spanish Flu pandemic of 1918–1919 waxed and waned over 2 years, evolving in the process, eventually reaching every corner of the planet and striking down an estimated 50–100 million people.^1^ Disproportionately, it killed young adults. There was no vaccine and no effective treatment. In many cases, the viral illness killed through secondary bacterial pneumonia. At that time, antibiotics to treat these secondary infections did not exist.

A century later, we are dealing with a different virus that attacks in a somewhat different way. Severe acute respiratory syndrome coronavirus 2 (SARS-CoV-2)—the virus that causes coronavirus disease (COVID-19)—infects the lungs directly and also causes death by inducing a state of “cytokine storm,” disturbing coagulation, and attacking other organs. Disproportionately, it kills the very old. The new virus appears to be similarly infectious^2^ and similarly lethal^3^ to the Spanish Flu. However, these are still early days and there’s much we do not know, including how it may affect different populations in different geographies and what long-term sequelae may result.

Modelers from Imperial College London and elsewhere have warned that, unimpeded, SARS-CoV-2 could kill millions over the next 1–2 years.^4^ Although it is appropriate that we focus very seriously on impeding progress of the pandemic, at the same time, we need to give equally serious attention to ensuring the least terrible collateral outcomes. And that will be tricky.

Policy makers and public health officials around the world are struggling to optimize their response across multiple dimensions of complexity: to maximize benefit and minimize collateral harm. One common denominator across settings is the virus itself. So, some of the transmission dynamics, for example, the virus’ incubation period, are fairly stable and predictable across all settings—both high-income and upper middle-income countries (referred to in this article as higher-income countries [HICs]) and low- and lower middle-income countries (collectively referred to in this article as LMICs). But many other parameters that are relevant to finding the best solutions vary considerably across settings*, so we need different strategies for different settings.*

## THE HIC BLUEPRINT

### Phase 1: Suppression

Although the details and relative emphasis across elements of the strategy have varied somewhat, nearly all HICs have responded to the COVID-19 epidemic using essentially the same paradigm, albeit with varying degrees of effectiveness. They began with an aggressive suppression phase, aiming to rapidly reduce the *effective reproductive number* (R_t_)* to less than 1. The popular media has described this more often using the less technical expression, “flattening the curve.” The key strategic goals of this phase have been to avoid overwhelming hospitals and to buy time, which has entailed ramping up capacity for resource-intensive critical care (notably ventilators and intensive care unit beds) to treat severe cases, and to conduct testing, tracing, isolating of identified cases and quarantining their close contacts to control spread. Achieving rapid reduction of R_t_ to <1 has required very substantial reduction in social contact, attained in most instances through shelter-in-place and lockdown policies. Across all HICs, it has been recognized that it was critical to get in place robust provisions for test-trace-isolate.

There has been some variation by country and region. Generally, in East Asia, there was a rapid initial response with aggressive provisions for test-trace-isolate (including institutional isolation and quarantine of cases and close contacts). As a result of rapid and generally pretty effective action, the cumulative incidence in these countries, to date, has been comparatively low. In countries in Europe and North America, in general, the initial response was slower (Germany being a notable exception). There has been less appetite for out-of-home, institutional quarantine; instead, most jurisdictions in these regions have used a strategy of population-wide lockdown.^5^

During the suppression phase in some HICs, certain socioeconomic arrangements, such as meat-processing plants in North America and migrant worker dormitories in Singapore and the Gulf states, have created super-spreading conditions where special efforts have subsequently been needed to bring transmission under control. In a number of HICs, the elderly living in nursing homes and assisted-living facilities have been at very high risk and have accounted for a large proportion of deaths.** Thus, these settings, too, have more recently been given focused attention to ensure a higher level of protection from exposure. *The original strategy had to be adapted based on facts on the ground*.

### Phase 2: Maintenance

Beyond the initial suppression phase, HICs are now beginning to transition to a maintenance phase. The goal in this second phase is to keep R_t_<1 (i.e., keep the daily count of incident cases on a downward trend), through continued, but less restrictive, physical distancing (more commonly described as “social distancing”) and provision for rigorous test-trace-isolate action, quickly identifying cases and outbreaks, and rapidly moving to contain them. The decision about how early to relax lockdown and begin moderating physical distancing is complicated and involves tradeoffs.

All else being equal, keeping the suppression phase in place for longer before transitioning to maintenance phase means reducing the levels of ongoing transmission considerably lower. But this level of transmission can only be achieved at high cost due to the disruptions caused by prolonged stringent lockdowns. Mitigating collateral harms during the suppression phase has been enormously costly, but because these countries have deep pockets, more or less adequate provisions for social insurance have been feasible (at least for now).

## BUT WILL THE HIC BLUEPRINT WORK IN LMICS?

LMICs differ from HICs not just in terms of available resources but also in having substantially younger age distributions. Most fragile and conflict-affected or post-conflict states fall into this LMIC economic category. The differences in resources, age structure, stability, and state capability are relevant to determining likely benefits, harms, and feasibility associated with possible responses to COVID-19. The challenge for decision makers is to choose feasible, contextually-appropriate strategies, balancing expected benefits versus harms and seeking to achieve the least terrible population outcomes.

In relation to efforts to control COVID-19 in LMICs, let’s consider 3 questions:

1. What is the expected benefit?
2. What harms could arise?
3. How feasible is the “flattening the curve” mantra?

### 1. What is the Expected Benefit?

#### Age Structure

As we have noted, unlike the Spanish Flu, which disproportionately ended the lives of young adults, COVID-19 is disproportionately killing the elderly and those with certain underlying morbidities; therefore, its impact varies by population structure. Around the world, there are marked differences between populations. In Canada, 17% of the population is aged ≥65 years; many European countries have more than 20% of the population aged ≥65 years and Japan has 28% aged ≥65 years. By contrast, most countries across South Asia and sub-Saharan Africa have between 3% and 4.5% in this age group (i.e., about 7-fold less as a proportion of the whole population).

Although COVID-19 is certainly causing some deaths among younger adults and mortality risk increases for those in their 60s and into their 70s, *risk is especially high for the very old.* In Canada, those aged 80 years or older account for only 4% of the population but for 70% of COVID-19 deaths.^5^ Using the age structures of Canada^6^ and Nigeria^7^ in 2018 and age-specific case fatality^8^—all else being equal—if 70% of both countries’ populations were infected, the expected number of deaths/100,000 in Canada would be 1,358; in Nigeria, 265—5-fold fewer. So, the expected mortality impact of the epidemic, unhindered, is considerably lower in most LMICs than in HICs^†^; therefore, the benefit of stringent control measures like sustained lockdowns is much less in LMICs than in HICs.

#### But There Doesn’t Seem to Be Much COVID-19 Yet in LMICs

What should we make of the apparently low number of cases, to date, in South Asia and sub-Saharan Africa? In part, we may be seeing the lull before the storm. Compared to Europe and North America, early in 2020, there was considerably less seeding of cases imported into most LMICs from other countries. Many LMICs imposed lockdowns, restricted flights, and closed borders comparatively early in the epidemic. In some LMICs (e.g., island states, parts of sub-Saharan Africa), initial spread may be impeded by less dense transportation linkages. And in some societies, because of the structure of social networks, spread may be slower (this may, for example, be the case in rural West Africa).^9^

A large proportion of cases may have minimal symptoms (due to age structure, and probably due to less obesity); more severe cases are either not getting to hospital or, if hospitalized, not recognized as cases of COVID-19. This would give an iceberg-like scenario, with most disease activity below the water line.

It has been speculated that temperature or other climate-related effects may mitigate the COVID-19 impact in the tropics, although evidence available to date^10^ doesn’t provide strong support for this hypothesis. Note that the Spanish Flu had especially crushing impact in South Asia, which may have suffered a quarter of all deaths (as many as 25 million, according to Barry^1^). Even if seasonality turns out to be a characteristic of COVID-19 (as it is for the commonly circulating coronaviruses), there is likely to be a muddier, more complicated seasonal picture in the tropics (as there is for influenza). There has also been speculation, based on work done on “innate immunity” and correlations at the country level, that prior exposure to BCG could confer some protection against COVID-19, resulting in less transmission in countries that have widely used this vaccine.^11^ This may give us some reason to hope; however, the available ecologically-grounded evidence is weak.^12^

But most important, to date there has been far less testing than in most HICs, so—to the degree COVID-19 is already spreading—it has been largely invisible (indeed in some countries it appears it has been deliberately kept invisible). Based on the most recent reported data^13^ available at the time this article was written *all* LMICs have been testing fewer than 1 person/10,000 population per day; some (Nigeria, notably) have been testing fewer than 1/100,000 (in some settings, the quality of testing has also been problematic). At this level of testing, we don’t know what’s really happening with spread of the virus.

### 2. What Harms Could Arise?

#### Counterproductive Effects on COVID-19 Spread

In most LMICs, strict lockdowns—particularly if efforts are made to sustain them beyond a few weeks—are likely to cause considerable harm. Indeed, even for the avowed purpose of reducing spread, some control measures have been counterproductive. For example, in some countries, lockdowns have resulted in chaotic, high-volume out-migration from cities,^14^ as informal-sector workers try to return to their home villages, carrying the virus with them. Similarly, hundreds of thousands of migrant workers have recently been obliged to return to their countries of origin—notably in South Asia—seeding new outbreaks on their return.

#### Impact on the Economy and Food Systems

In almost all LMICs, the majority of the working-age population is employed in the informal sector. Compared to HICs, most LMICs have weaker capacity and much fewer resources to draw on to provide adequate social insurance measures to support those whose employment is interrupted due to control efforts. With overly aggressive control measures, half a billion people risk being pushed into absolute poverty.^15^

Disruptions to food systems that impair food production and distribution (e.g., through border shutdowns and suspension of trade in sub-Saharan Africa, where many countries depend on imports for a large proportion of their food) risk creating mass hunger, possibly compounded in some regions by locust infestations or drought.

#### Lack of Access to Education

Education of children is a key concern in all societies, but even more so for countries with young populations, recognizing that education is a vitally important determinant of future life opportunity. Online education is not an option for the overwhelming majority of families in LMICs. Sustained disruption of schooling will have serious long-term consequences, especially for the poor. Furthermore, given that severe COVID-19 illness is rare in children, that they appear to be less susceptible, and available evidence that child-to-adult transmission has played a very small role in propagating the epidemic,^16^ the epidemiologic rationale for keeping schools closed is weak.

#### Disruption of Routine Clinical Services

In HICs, there has been a major focus on trying to ensure adequate critical care beds for the expected burden of severe COVID-19 cases. However, most LMICs cannot realistically expect to provide such care for all who could benefit from it (though they certainly need to be making efforts to provide some degree of care for such cases, to the extent possible^‡^). Disrupting services and discouraging people from seeking care in hospitals can be expected to reduce timeliness of treatment for potentially life-threatening conditions like appendicitis and myocardial infarction. Indeed, in some LMICs, there have been anecdotal reports of private hospitals refusing to admit any patients with fever or other symptoms suspicious for COVID-19. In addition to discouraging timely recourse to hospital services for acute medical problems, heavy control efforts risk undermining pharmaceutical supply chains and impairing access to drugs needed by those with significant chronic medical problems like diabetes and hypertension.

#### Reductions in Use of Primary Health Care

Based on their modeling, investigators from Imperial College London^17^ estimate that COVID-related disruption to tuberculosis and HIV services could result in a 10% increase in HIV mortality, mostly as a consequence of interruptions in antiretroviral treatment.^14^ With current annual HIV mortality estimated at 770,000,^18^ that translates to 77,000 excess deaths over the coming 12 months. The Imperial College investigators estimate that disruption to tuberculosis services could result in a 20% increase in deaths, mostly due to reductions in timely diagnosis and treatment of new cases. With 1.5 million^19^ people currently dying from tuberculosis per year, that would mean 300,000 excess deaths over the coming year.

Routine clinical preventive services may not seem glamorous, but when they are allowed to lapse due to suspending outreach services or absence from post by health workers afraid of contracting the infection, this will quickly result in increases in otherwise easily preventable deaths. Significant disruptions to malaria services (notably distribution of insecticide-treated nets and access to treatment services) could result in a 20% increase in malaria deaths (mainly among children age <5 years in Africa), an excess of 80,000 over the coming year.^20^

From their modeling work using the Lives Saved Tool, investigators from Johns Hopkins Bloomberg School of Public Health^21^ estimate that if there are significant enough disruptions to maternal, newborn, and child health programs at the primary health care level in LMICs to reduce coverage for key interventions by 40%–50%, there would be an excess of 1,157,000 child deaths and 56,700 maternal deaths *over the next 6 months* (i.e., almost 4 times as many as deaths directly caused by COVID-19 worldwide over the 6-month period from when the epidemic began through mid-May (318,000 deaths at time of writing). Is a death due to COVID-19 necessarily a bigger tragedy than the death of a 3-year-old that would not otherwise have occurred (or 4 such excess deaths, for that matter)?

#### Burden of Vaccine-Preventable Diseases

From 1988 to 2010, due to effective efforts in LMICs to ensure that all pregnant women are vaccinated against tetanus, the number of deaths per year due to neonatal tetanus decreased by 93%, from an estimated 787,000 to 58,000.^22^^,^^23^ Significant reductions in tetanus immunization of pregnant women due to interruptions in use of routine antenatal services would result in a corresponding rise in newborn tetanus deaths.

Polio has been on the point of eradication. With suspension of control efforts, there is every reason to believe it will silently spread, setting back eradication efforts by years. In Afghanistan, Nigeria, and Pakistan, polio vaccination campaigns have stopped.

Measles is an extremely contagious disease which, in addition to killing through direct infection, results in the infected child having reduced immunity for months after the initial infection (analogous to AIDS) with a large proportion of deaths due to secondary infections. In poor countries, measles kills 3%–6% of those it infects (mostly infants). With major gains in measles immunization coverage, measles was, for a time, eliminated from the Western hemisphere, but now it’s back. In 2018, there were 140,000 deaths attributable to measles.^24^ As recently as 2000, there were close to 1 million measles-associated deaths worldwide among young children (60% in sub-Saharan Africa and 30% in South Asia). In the pre-immunization era, there were far more measles deaths. With an extended disruption of immunization services, there will certainly be major measles outbreaks in LMICs. There is certainly reason to fear hundreds of thousands of additional measles deaths over the next 1–2 years. The greater the disruption to routine immunization activities, the greater the number of such deaths.

With this many lives at risk, how much disruption can we afford?

### 3. How Feasible is the “Flattening the Curve” Mantra?

We have already noted much younger populations in LMICs than in HICs. There are also marked differences in household structure and associated degree of social contact by age category.^4^ In LMICs, social contact rates are similar across age groups and the majority of the elderly live in multigenerational households, typically numbering 5 or more people. By contrast, in most HICs, average social contact rates decline considerably from mid-adulthood on and most of those aged 65 years or older live either alone or as couples (although a significant proportion of the very elderly, aged 80+ years, live in nursing homes and assisted-living facilities). This has consequences for social mixing and the opportunity for spread to these high-risk older people.

Related to number of household members, there is also the issue of floor-space per person. This varies directly with per-capita income, with much less space per person, on average, in LMICs than in HICs. Tighter quarters means enhanced opportunity for transmission. Of course, this is true not only within households but also within communities. Housing density varies markedly by setting. At the extreme end of the spectrum are major slums in LMICs; a good example is Dharavi, a slum in Mumbai numbering 1.2 million living in an area of 2.4 km^2^ (500,000 people/km^2^). In such settings, we have multiple factors compounding the likelihood of spread: within-household crowding, within-neighborhood crowding, many homes lacking direct piped water, a large proportion of the working-age population engaged in the informal sector, and generally weak state capability and governance.

Consider the following:

- Self-isolation has been promoted in HICs for those with either new respiratory symptoms or recent contact with a possible case. At population-wide scale in most LMICs, how feasible is this likely to be?
- In all countries, efforts have been made to suspend planned mass gatherings. In countries with comparatively weak state capability or distrust between the State and the population, or sensitivities about interfering with religious activities, how feasible will it be to maintain such proscriptions?
- In settings where people depend on informal (sometimes crowded) markets to procure their food on a daily or near-daily basis, how feasible will it be to close them down?
- How long can hard lockdowns be sustained in settings where most people work in the informal sector?
- How likely is it over the short- to medium-term, in most LMICs, that there will be adequate capacity for robust, widespread testing, contact tracing, and effective isolation of identified cases and their close contacts?
- On the positive side, many societies in LMICs have very robust social systems that represent an important resource to be drawn on in response to COVID-19, although they are currently quite constrained by hard lockdowns.

It is certainly possible that Sub-Saharan Africa and South Asia may represent less fertile ground for the virus. At this point, we really don’t know. But, with all of these challenges, how realistic is it to think that R_t_ can be reduced to and maintained at less than 1 in most LMICs? Even some HICs are currently struggling to achieve reductions in new cases (evident in some Latin American countries at the time this article was written).

## IF THE HIC BLUEPRINT ISN’T A GOOD FIT, WHAT WOULD MAKE MORE SENSE?

Yes, everyone is preoccupied with risk associated with the infection, but Jones^25^ has challenged us also to take into account the “risk of exaggerated fears and misplaced priorities.” Decision makers in LMICs need to make strategic choices prioritizing across multiple domains, taking into account demographics and resources available, and considering: what’s feasible, what’s acceptable, and what’s the expected net benefit/harm of the choices being considered. As we have been arguing, the 2-phase approach pursued in HICs with the goal of initial rapid suppression—pushing R_t_ to less than 1— and then moving into a maintenance phase, keeping incidence low, is unlikely to be feasible in most LMICs. Instead, LMICs will need to find ways of coping with a continued rise in incidence and minimizing overall negative impact. Certainly, other important issues will need attention (notably, management of severe cases). In most if not all LMICs, 3 key objectives will likely be appropriate:

1. Limit spread, short of full suppression
2. Mitigate harms associated with control efforts
3. Shield those at high risk (and avert deaths)

Let’s consider each of these, in turn.

### 1. Limit Spread

If it’s accepted that with the efforts that can feasibly be deployed on a sustained basis (i.e., for many months) it will not be possible in most LMICs to push R_t_ to less than 1 and keep it there, the number of new cases will continue to increase. This means continued spread until the virus runs out of susceptible hosts to jump to and further spread spontaneously fades off—the level popularly described as “herd immunity.”

Accepting continued growth in new cases does not mean efforts should not be made to slow the increase. Even if an R_t_ of less than 1 cannot be achieved and maintained, a slower increase means a smaller volume of severe cases at any given time and fewer infected over the long term. Key elements of a strategy to slow spread should include:

- Recommending locally-appropriate, sustainable forms of physical distancing, including: encouraging universal mask use when in prolonged close contact in tight, enclosed spaces, for example on buses and trains (as well as masks and other personal protective gear for health workers); installing barriers made of plexiglass or similar material to protect workers in close contact with customers or co-workers; shifting activities outdoors where feasible; encouraging and supporting appropriate respiratory etiquette (covering coughs/sneezes; into inside of elbow, not hand) and hand hygiene (improving access to water, to the extent feasible); practicing self-isolation for those who have new respiratory symptoms.
- Eliminating or at least reducing super-spreading circumstances using feasible, locally-appropriate, solutions including seeking to suspend mass gatherings and decongest crowded conditions in prisons, slums, barracks, migrant-worker dormitories, markets, and workplaces. In all informal urban settlements, there need to be emergency planning committees formed to work out effective, feasible, and acceptable solutions.
- Instituting as robust provisions for test-trace-isolate are feasible, strategically targeting available testing capacity, quarantining confirmed cases and their close contacts, and identifying and controlling outbreaks. In principle, there is potential for big impact on spread if this is done widely and well (and this is much less disruptive than population-wide lockdowns).

### 2. Mitigate Harms

As we have noted in the earlier discussion, with more extreme control efforts there is every reason to believe there will be more severe consequences (already evident in many places). With a high proportion of the population working in the informal sector, generally quite limited scope for social insurance support for those unable to work, and high risks for widespread impoverishment, mitigating such harms requires less aggressive disruption of the economy. Food systems need to remain functional. Health care, including routine clinical preventive services, needs to be maintained with minimal disruption, through more targeted, less draconian approaches to physical distancing.

As in HICs, attention in LMICS also needs to be directed toward mitigating potentially serious psychosocial consequences including anxiety or depression, stigma, sociopolitical unrest, and violence that may arise from people’s response to the epidemic and associated control efforts. In part, mitigating such consequences can be achieved through sound, strategic communication to health workers and the general population^26^: having a trusted, authoritative source of information; disseminating accurate, useful information; preemptively addressing fear and anxiety, myths, and misinformation; and encouraging support for the vulnerable. There needs to be honesty about the facts: many will be infected, and this won’t be over soon.

### 3. Shield Those at High Risk

Those most at risk are the elderly.^§^ In LMICs, most of the elderly live in multigeneration households, not on their own or in institutions (as is more common in HICs). Protecting the *shielded* will require reducing the networks of possible transmission into the home and to the elderly members of the household, by mobilizing members of the household and others as *shielders*, who will need to adopt more rigorous physical distancing practices.^27^ In crowded living conditions, this will be difficult. Effective strategies need to be developed locally.

The best protection would, of course, be an effective vaccine. It may well be that effective (and affordable) treatments will be identified over the coming year and beyond, in which case, their use can also form an important part of the response. As we wait for that moment, world leaders need to commit that when such a vaccine is first available, priority for its distribution will be based on *need* (notably the elderly and those with relevant chronic conditions) not *wealth*.^28^

## SOME FINAL WORDS

We are certainly not the first to note the problem of uncritical mimicry of HIC solutions in response to the COVID-19 epidemic^29^^,^^30^ or to suggest that different strategies are needed in LMICs.^28^^,^^31^^,^^32^ But the needed new strategies are not yet much in evidence. We call on national governments and the international agencies and donors seeking to support them to undertake the tough challenge of crafting context-appropriate strategies that appropriately balance expected benefits, potential harms, and feasibility. Such strategies are likely to look quite different from what we’ve seen, to date, in HICs. As frightening as this virus itself may seem, if it commands all of our attention to the exclusion of broader considerations, misplaced priorities could result in greater harm.

## Footnotes

* The *basic* reproductive number, R_0_, is the average number of new incident cases arising from each infection in the absence of any control measures. The *effective* reproductive number, R_t_, which we focus on in this article, varies over time depending on control measures.

** Indeed, in some HICs, like Canada, preemptive efforts to decongest acute care hospitals to accommodate an expected surge of COVID-19 cases resulted in more dangerous super-spreading conditions in many of the long-term care facilities into which these patients were transferred and, ultimately, in higher numbers of COVID-19 deaths than would otherwise have occurred.

† Clearly, factors other than age can contribute to risk of severe disease. Nutritional factors and co-existence of certain other infections may increase risk in LMICs. Furthermore, for those experiencing more severe COVID-19 infections, case fatality is likely to be higher in the absence of quality critical care services. It may certainly turn out to be the case in many LMICs that case fatality is higher in younger age groups than what has been seen in East Asia, Europe, and North America.

‡ Even if intensive care unit beds and ventilators are not available, other elements of care can be provided to improve survival and reduce morbidity among severe, hospitalized cases. Efforts to make oxygen much more widely available in secondary-level health facilities (health centers, district hospitals) would certainly be helpful. And certainly, as in HICs, efforts need to be made to improve infection prevention procedures and practices and to ensure health worker safety.

§ Certainly the same principle of shielding applies also for those with important underlying conditions like chronic heart or lung conditions, poorly controlled diabetes, obesity, or compromised immunity, who are also at elevated risk of severe infection.
